# Update of Argentina’s Regulatory Policies on the Environmental Risk Assessment

**DOI:** 10.3389/fbioe.2021.834589

**Published:** 2022-01-31

**Authors:** Facundo Vesprini, Agustina Ines Whelan, María Florencia Goberna, Mariana Lucía Murrone, Gabriela Evangelina Barros, Andrés Frankow, Perla Godoy, Dalia Marcela Lewi

**Affiliations:** National Directorate for Bioeconomy, Ministry of Agriculture, Livestock and Fisheries, Buenos Aires, Argentina

**Keywords:** GM crop, data transportability, stacked GM crops, unintended effects, history of safe use, environmental risk assessment, familiarity, insects resistant management plan

## Abstract

The Environmental Risk Assessment (ERA) of genetically modified (GM) crops in Argentina is carried out by the National Advisory Commission on Agricultural Biotechnology (CONABIA) and the Innovation and Biotechnology Coordination (CIyB). Both have a large experience with this assessment, since 1991, when CONABIA was created. The continuous support to biotechnology as a state policy and as part of the decision to encourage developers in the regulatory process has helped make progress in the revision of the regulations. The experience gained during the last 30 years and the worldwide scientific advances supported the bases to update the regulatory framework. Focusing on the biosafety strengthening and the improvement of the applicant’s experience in the GM crops evaluation process, during 2020 and 2021, the ERA went through a reviewing process. Some important modifications were made, such as (i) the assessment of stacked GM crops with focus on the possible interactions between transgenes and the expression products, (ii) the strengthening of the ERA taking into account the transportability of data and conclusions from the Confined Field Trials (CFTs), (iii) the adoption of Familiarity and History of Safe Use (HOSU) concepts on the risk assessment of the expression products, (iv) the special considerations for the unintended effects of insertional sites, and (v) as a post commercial release of GM crops, the Insect Resistance Management Plan (IRMP) was reformulated. These novel approaches enhance the ERA; they make it more efficient by applying the science criteria and the accumulated experience and scientific bibliography on the topic.

## Introduction

Argentina was one of the first countries to have a regulatory framework for genetically modified (GM) crops for agricultural use. The evolution of the Environmental Risk Assessment (ERA) in Argentina is based on the updating of the regulations for different activities with GM crops as science advances and the experience accumulated. Argentine regulations have been in force and running since the early 1990s and take into account the criteria and considerations established in the Cartagena protocol and other international treaties. At the time that the National Advisory Commission on Agricultural Biotechnology (CONABIA) and the Innovation and Biotechnology Coordination (CIyB) decided to work on updating the regulations that contemplate the requirements for the commercial authorization of GM crops from the environmental point of view of agro-ecosystems, different issues involved in the risk assessment were identified. These different issues were considered and treated in order to simplify the regulatory process of these products. It should be noted that the main aim in the updating process is biosafety. The purpose of this paper is to describe the evolution of this process and how the regulations for different topics were developed and updated. These topics were data transportability, stacked GM crops, Familiarity and History of Safe Use (HOSU), unintended effects, and Insect Resistance Management Plan (IRMP). Despite the fact that assessment by similar constructions is not described in this review, the criterion was ratified. It is based on GM crops with similar constructions that share the same characteristics of interest using the same molecular mechanisms to other commercial GM crops. The assessment criterion is based on establishing the absence of new or increased risks with respect to the previously assessed GM crop. Additionally, risk assessments are framed from the application of an analysis system based on the Problem Formulation (PF). Under this consideration, risk hypotheses that are identified linked the crop, the new phenotype, and its interaction with the agro-ecosystem, with focus on biosafety.

## Updated Processes on Regulatory Policies

### Assessment of Stacked GM Crops

Stacked GM crops refer to conventional breeding crossing single GM crops containing individual transgenes with single or multiple traits. Single GM crop is defined as the insertion of DNA into the plant genome as a result of a single transformation process (Pilacinski et al., 2011). Many of the stacked GM crops contain insect and herbicide tolerance traits for controlling a broad range of insect pests and weeds (Que et al., 2010). Each single GM crop must have gone through the ERA and have a safety conclusion to apply at the stack assessment.

In the beginning of the stacked GM assessments, each application was considered as a new GM, and it went through the full assessment as a single GM crop. Therefore, all the molecular, phenotypic, and the interaction between the stack GM crop and the environment had to be presented. With the accumulated experience and based on the problem formulation approach, referring to analyze and verify risk hypotheses considering the weight of evidence, the assessments have gone through a simplification process, where redundant information related to each single GM crop was left aside. Using conventional breeding to combine GM crops does not involve insertion of new recombinant DNA sequence into the genome and does not modify the existing genomic DNA (Pilacinski et al., 2011).

From the above review process, applying the PF approach and considering the case-by-case assessment, the CONABIA and the CIyB decided that the assessment of stacked GM crops must focus on the possibility of interaction between novelty traits and genes. It was one of the most relevant topics of the resolution 32/2021 from the Secretary of Food, Bioeconomy, and Regional Development. The potential of interactions in the stacked GM crops is based on an understanding of the mode of action of the transgenes and their products (Kramer et al., 2016), specifically the possibility of epistasis between introduced genes or interaction between expression products in related metabolic pathways. At the same time, specific data to verify the absence of interaction, when supported by a risk hypothesis, became relevant, for example, to verify the absence of synergism between insecticide proteins. If it exists, a new non-target organism study must be done with the combinations of proteins. As a result of this interaction assessment, the risk for the environment of planting the stacked GM crop is analyzed.

### Transportability of Data and Conclusions From the Confined Field Trials

In the ERA for the commercial release of GM crops, the CONABIA and the CIyB have applied some complementary approaches about the transportability of data and conclusions from CFT. CFTs are based on a comparative agro-phenotypic assessment between transgenic and non-transgenic (usually the isogenic or a near-isogenic line) with the aim to identify any differences between the GM crop and its non-GM comparator resulting from the intended or unintended consequences of the genetic modification (García-Alonso et al., 2014; Nakai et al., 2015). With these data, risk hypotheses are answered. CFTs involve plants grown side by side that are therefore subject to the same environmental conditions and agronomic management (Vesprini, et al., 2020). Data transportability builds on the premise that well-designed CFT may inform the ERA and support regulatory decision-making for GM plants being cultivated in another country (García-Alonso et al., 2014; Ahmad et al., 2016; Vesprini et al., 2020).

In the beginning of the Argentinean ERA, local CFTs were required and studies from other countries were considered as a weight of evidence to support the conclusion about the GM crop biosafety. Later, as García-Alonso et al. (2014) describes, foreign CFT replaced some local ones if they were done in similar agro-ecological conditions as the Argentinian crop production zone. This approach of data transportability comparing similar environments (climate, weather, and soil type) between regions to transport data became a useful tool to avoid redundant CFTs. At the same time, if the CFT is replicated in the country of interest, it is expected to have the same conclusion.

After years of ERA, it was evidenced that the conclusions arrived at in CFT that were analyzed in a wide range of environmental conditions can be transportable to other geographies, regardless of the agro-climatic and agro-ecological conditions (Vesprini et al., 2020). On this approach, the site selection with focus on the diversity of tested environments examined were key elements (Vesprini et al., 2020). The diversity selection of environmental conditions to perform the CFT is related to the crop production zone. At the same time, as the approach comparing agro-ecological conditions, the methodology and agronomic management of the studies and the measured endpoints are relevant to consider (Vesprini et al., 2020). If these three items are met, not only the data (as an informative study) but also the conclusions of the CFT are transportable. Therefore, if this study is performed again considering other wide environments of the crop production zones, the conclusions arrived at will not change. This approach has specific considerations for risk hypotheses obtained through PF related to the GM crop and its interaction with specific environments. If any of these risk hypotheses needs a CFT in the site of interest, performing the CFT in that site may be justified. Otherwise, comparing agro-ecological conditions is a useful tool to analyze if the environmental conditions under concern were considered in a foreign CFT.

### Familiarity and History of Safe Use

The CIyB and the CONABIA have carried out numerous ERA of different GM crops, repeatedly evaluating the same expression products. Moreover, both in Argentina and in many other countries, the commercial cultivation and consumption of crops expressing these expression products provide HOSU and support the conclusions reached by the CIyB and the CONABIA in the decision documents.

Recently, the CIyB updated the risk assessment process for GM Crops based on the familiarity and history of safe use (HOSU) of crops and expression products.

The concepts of HOSU are an integral part of PF, as the availability of existing information is a critical element that adds to the weight of evidence (Capalbo et al., 2020).

Familiarity is defined in the new Argentine guideline as “pre-existing scientific knowledge, experimental evidence, and accumulated regulatory experience on new expression products or on GM crops that can be taken into account in an ERA”. Thus, the collection of documents, data, and existing literature constitute support material and form the weight of evidence for ERA.

Additionally, the HOSU is defined in the new Argentine guideline as the tradition in use, where scientific procedures or formal knowledge are not necessarily available or limited. However, given the history reported by the empirical evidence of use without adverse effects, it can be used as strong evidence to reach conclusions about the safety of new expression products, GM crops, or receptor crops. Both definitions have been supported in Capalbo et al. (2020).

By applying these two concepts, the main goal is to avoid redundancy of information declared in the different ERA applications.

All in all, in the new guideline, the applicant has the option to report if the expression products have familiarity or HOSU. In the event of no new or different information having emerged in relation to previous ERA performed for those expression products, it will be considered that the product has familiarity and/or HOSU.

However, it should be noted that, since the analysis is done on a case-by-case basis and is based on scientific/technical reviews, this measure will be ineffective if there is new information that invalidates the conclusions on which the previous opinions were based. Therefore, new relevant information must be presented and submitted to the CIyB and the CONABIA for consideration in order to carry out the analysis.

### Unintended Effects of Insertional Sites

The CIyB and the CONABIA also updated the ERA for GM crops related to the unintended effects of insertional sites. During the genetic engineering transformation process, the DNA fragment of interest is inserted into the genome of a plant, often accompanied by additional DNA fragments and can also generate deletions and/or rearrangements. These genetic changes are collectively known as insertion effects and have the potential to give rise to unintended traits in plants (Schnell et al., 2015). These modifications could also alter genes or regulatory elements of the plant genome and generate new open reading frames (ORFs).

The relationship between genotype and phenotype in plants is complex and the role of the environment cannot be ignored. In many ways, plants are buffered against the consequences of genomic changes by the high level of genetic redundancy in their genome and by the quality control systems active in them. All of these factors influence whether or not an insert effect will produce an unintended characteristic (Schnell et al., 2015). Moreover, plant genomes are very dynamic, plastic, and undergo frequent insertions and other rearrangements (Ladics et al., 2015).

Glenn et al. (2017) conclude that extensive regulatory requirements have been established for GM crops, using a comparative safety assessment process. Thereafter, numerous studies have found transgenic varieties to be compositionally equivalent to conventional crops and that there are few exceptions of cases where the desired trait confers an intentional change in composition, such as improved nutrition. Moreover, the above-mentioned author states that global GM crop regulators have concluded over the past 20 years that, excluding GM crops with an intentionally improved composition, all evaluated traits of commercialized GM crop varieties are equivalent to varieties with a history of safe use. This is, in part, the result of the same plant selection practices used by breeders to minimize unintended effects, whether arising from spontaneous genetic changes that occur during conventional breeding (Schnell et al., 2015) or from the use of biotechnology to insert DNA into the plant genome.

Both the new ORFs and flanking sequences could be analyzed by the data generated in the field assays. When these effects appear, the plants are discarded by the developers during the screening process of the different events, in the field, in the greenhouse, or in the laboratory (Privalle et al., 2012; Glenn et al., 2017). This way, the absence of unintended effects is confirmed by an adequate formulation of the risk hypothesis of the GM crop on the agro-ecosystem, and is answered by carrying out the agro-phenotypic characterization studies that include the analysis of different parameters such as germination power, seed latency, phenology, phenotype, and behavior against biotic and abiotic stresses, which are carried out in multiple sites that cover a wide variability of agro-climatic conditions.

Bearing these considerations in mind, the updated guideline only refers to the unintended effects caused by the insertional site, but other effects caused by different mechanisms are not considered. Additionally, the compositional analysis is always exhaustively assessed by the Food and Feed Safety Committee (CTAUOGM) from the National Service of Agri-Food, Health and Quality (SENASA).

Taking this into account, the applicant has the option to complete a form explaining the unintended effects in relation to the risk of the GM crop on the agro-ecosystem, according to what has been observed in the agro-phenotypic studies.

### Insect Resistance Management Plan

Evolution of resistance in insect populations is a natural process and agricultural practices are intended to delay or mitigate insect resistance management (IRM). The Argentine experience through the past years has shown that joint actions must be taken by all parties involved such as industry, growers, and governmental agencies (Signorini et al., 2018). One of the key measures for delaying the evolution of resistance is the implementation of a refuge area in a GM insect-resistant plot in addition to crop rotation, weed management, insect monitoring, and insecticide applications when pest populations reached economic thresholds in refuge and communication programs about the topics above.

The previous guideline (2014) was updated with several recommendations so as to improve this plan for the applicants and for the regulatory system. The changes made were as follows:

1. The IRPMs are presented only for those GM crops that are going to be commercialized (single or stack) in such a way to avoid unnecessarily presentations and information.
2. Optional models can be introduced. The computerized model gives information about the product life cycle. The percentage of refuge can be justified by other means, for instance, papers and other documents showing crops with the same proteins and pests.
3. Specific decision document for IRPM is concluded when the plan is evaluated and the agreement is given by CONABIA and CIyB previously to enroll the GM crop in the National Registry of Cultivars from the National Seeds Institute.
4. The results of the susceptibility baseline must be presented together with the IRPM, and those of the damage baseline must be presented within 2 years from the date of registration of the first cultivar in the National Registry of Cultivars.

## Conclusion

During the ERA process, carried out by CONABIA and the CIyB, the PF methodology is applied, through the formulation of risk hypotheses of the GM crops on the agro-ecosystem. When this assessment concludes, a decision document with all relevant information of the analysis process is drawn up. This document reflects the conclusions on biosafety for the agro-ecosystem of the evaluated GM crops.

After 30 years of having started the regulatory path of GMOs (October 1991) and 25 years after the first approval of a commercial crop, Argentina has maintained a continuous process of improvement. The regulatory system has been proactive and dynamic, analyzing the dossiers on a case-by-case basis, based on science and maintaining high biosafety standards. This experience allowed CONABIA to be named as FAO’s reference center in Biosafety since 2014. Following the path of continuous updating and improvement and addressing the new challenges that arise, a specific regulation for molecular farming is currently being addressed and other topics related to biosafety in different subjects such as plants, animals, and microorganisms for agriculture purposes.

## Data Availability

The original contributions presented in the study are included in the article/Supplementary Material. Further inquiries can be directed to the corresponding author.
